# Renewable Cyclopentanol From Catalytic Hydrogenation-Rearrangement of Biomass Furfural Over Ruthenium-Molybdenum Bimetallic Catalysts

**DOI:** 10.3389/fbioe.2020.615235

**Published:** 2020-12-18

**Authors:** Shihang Meng, Yujing Weng, Xiaolong Wang, Hongxing Yin, Zhenfei Wang, Qi Sun, Maohong Fan, Yulong Zhang

**Affiliations:** ^1^College of Chemistry and Chemical Engineering, Henan Polytechnic University, Jiaozuo, China; ^2^Henan Key Laboratory of Coal Green Conversion, Henan Polytechnic University, Jiaozuo, China; ^3^Departments of Chemical and Petroleum Engineering, School of Energy Resources, University of Wyoming, Laramie, WY, United States; ^4^School of Civil and Environmental Engineering, Georgia Institute of Technology, Atlanta, GA, United States

**Keywords:** cyclopentanol, rearrangement, hydrogenation, bimetallic catalyst, furfural

## Abstract

Biomass furfural-like compounds are chemicals that cannot be extracted from fossil materials, through which a large number of fine chemicals and fuel additives can be opened up, but one big efficiency problem during the transformation is the accumulation of oligomers. Here, we propose a novel and efficient Ru-Mo bimetallic catalyst for selective hydrogenation-rearrangement of furfural-like compounds. The result showed that an unprecedented rearrangement product selectivity of 89.1% to cyclopentanol was achieved under an optimized reaction condition over a 1%Ru−2.5%Mo/CNT catalyst reduced at 600°C. Subsequent characterization suggested that the catalyst presented with weak acidity and strong hydrogenation activity for the reaction, which not only ensures the smooth hydrogenation-rearrangement reaction but also inhibits the accumulation of furan polymers. These findings provide a convenient strategy to tune the catalytic performance of Mo-based catalysts by controlling the reduction and carburization conditions, which appear to be versatile for the rearrangement of furans and similar compounds.

## Introduction

Nowadays, petrochemicals act as the largest driver of global oil consumption, but problems relating to the reserves and environmental pollutions are challenging. The shift from traditional fossil to renewable carbon resources such as biomass can decrease chemical production from fossil resources and CO_2_ emissions (De et al., 2015; Liao et al., 2020). Furfural (FFA) is widely identified as a key platform molecule for catalytic upgrading of biomass-derived feedstocks into useful fuels and chemicals because of its multifunctional characteristics, consisting of carbonyl (C=O) and Π-conjugated (C=C-C=C) groups in a five-membered ring structure, plus its current commercial-scale production from C_5_ sugars (over 280 000 ton per year) (Deng et al., 2018). Currently, furfuryl alcohol (FA), tetrahydrofurfuryl alcohol (THFA), and 2-methylfuran (MF), each containing a five-membered heterocyclic ring with one oxygen atom, are typical products of the selective hydrogenation of furfural. However, selective rearrangement of furfural into alicyclic five-membered carbon rings remains to be explored (Yang et al., 2013).

**Scheme 1:**
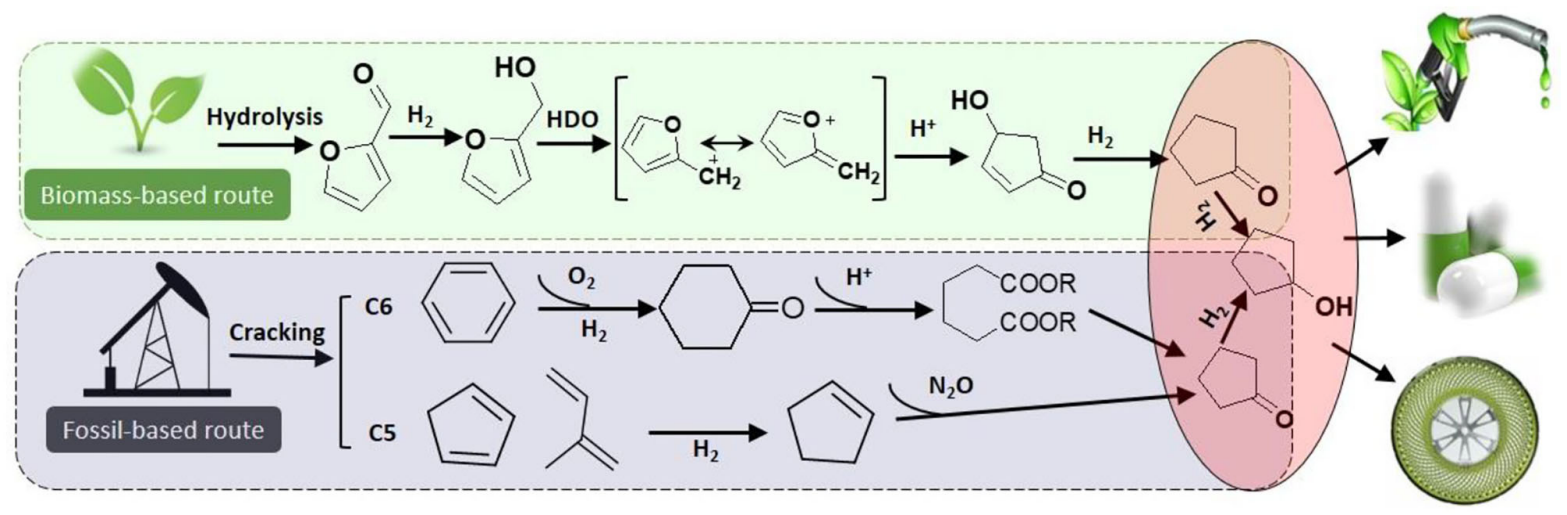
Routes to cyclopentanol from fossil oil by the C5 and C6 transformation, and in biomass furfural by the proposed process.

Cyclopentanone (CPO) and cyclopentanol (CPL) are fine chemical and raw materials used in the chemical industry. For instance, cyclopentanol is the simplest member of the class of cyclopentanols bearing a single hydroxy substituent, which are used for perfumes, chemical solvents, dyes intermediates, pharmaceuticals, and other organic products (Zhang et al., 2016). Additionally, natural cyclopentanol only exists at low concentrations in a small number of plants, which makes extraction difficult. Generally, cyclopentanol is produced by hydrogenation of cyclopentanone, while cyclopentanone is traditionally produced by decarboxylation of adipic acid or oxidation of cyclopentene (using fossil-based feedstocks), which is inefficient and polluting (Scheme 1). In contrast, the production of cyclopentanol from biomass-derived furfural presents an efficient and green approach.

However, challenges in the biomass route of furfural conversion still remain. First, side reactions, such as decomposition into small oxygenated compounds, over-hydrogenation, and over-hydrogenolysis easily occur. Second, the accumulation of oligomers on the catalyst surface (especially at acidic centers) from the condensation reactions between furans and reaction intermediates lead to rapid catalyst deactivation. Thus, selectivity to rearrangement products is very sensitive to the balance of metal and acid functions. To date, precious metal catalysts (Ru, Pt, Au, and Pd) and Cu supported on Lewis acid carriers (Al_2_O_3_, ZrO_2_, TiO_2_, and Co_3_O_4_, etc.) have been reported with valuable cyclopentanone yields, but the high selectivity of CPL is still challenging (Yang et al., 2013; Fang et al., 2015; Li et al., 2015; Hronec et al., 2016; Liu et al., 2016; Wang et al., 2016; Zhang et al., 2016, 2019). Among these, the Cu-based catalysts (Cu/ZnO_2_, Cu/Co_3_O_4_, etc.) have attracted significant interest due to their advantages of relative low hydrogenation activity and low cost (Yang et al., 2013; Guo et al., 2014; Li et al., 2015; Wang et al., 2016; Gong et al., 2018). However, the main issues are copper's low hydrothermal stability due to the loss of Cu^+^, Cu particle sintering, and poisoning of active Cu sites by adsorption of reduction intermediates, and coke formation/deposition (Chen et al., 2018), rendering it impractical to improve the catalyst performance in the hydrothermal environment.

Bimetallic combinations with oxophilic metals or additives are often elegant ways to provide multi-active sites to accomplish the coupled multiple reactions of furfural rearrangement (Chen et al., 2018). Recently, we reported the selective hydrogenation-rearrangement of furfural to CPL over bimetallic ruthenium-molybdenum catalyst, which obtained 74% carbon selectivity to CPL under the optimized reaction condition (Weng et al., 2018b; Wang et al., 2019). Herein, we extend our preliminary work, and present a detailed study on the screening of bimetallic catalysts and optimization of reaction conditions, especially, the proportion of bimetallic components. It was found that the catalyst with low metal loading had better catalytic performance in the rearrangement of furfural to CPL, among which the yield of CPL could reach as high as 89% under the optimized reaction condition (180°C and 4 MPa) over 1%Ru−2.5%Mo/CNT (reduced at 600°C). Subsequently, structural characterizations suggested that reduction in temperature could be used to tune the extent of reduction and carburization degree of Mo species, which resulted in different catalytic performance.

## Experimental Section

Furfural (C_5_H_4_O_2_) was supplied by shanghai Macklin Biochemical Co., Ltd. (Shanghai, China). Ruthenium-chloride-hydrate (RuCl_3_·3*H*_2_O) was purchased from Aladdin Co., Ltd. (Shanghai, China). Ammonium molybdate ((NH_4_)_6_Mo_7_O_24_) was purchased from Tianjin Fuyu Fine Chemical Co. Ltd. (Tianjin, China). Multi-walled carbon-nanotubes (CNT, inner diameter 2–5 nm, length 10–30 μm) was purchased from Chengdu Organic Chemicals Co., Ltd. (Sichuan, China).

Here, Ru-Mo/CNT catalysts were prepared by a sequential impregnation method reported previously (Wang et al., 2019). Firstly, a certain amount of carbon nanotubes were immersed into an ammonium molybdate aqueous solution, followed by ultrasonic treatment during the impregnation process. Then, the derived mixture was dried overnight in an oven, transferred into a quartz tube furnace, and calcined in a nitrogen gas flow (20 ml/min) at 400°C for 2 h. The calcined sample was immersed into an aqueous RuCl_3_ solution for Ru loading, similar to the impregnation of Mo. Finally, the obtained mixture was dried at 393 K overnight and then reduced in the quartz tube furnace under a mixture gas flow (Ar/H_2_ =9/1, 20 ml/min) for 4 h at different reduction temperatures (500–800°C at 100°C increments). All catalysts were passivated under an industrial nitrogen gas flow prior to the catalytic testing and characterization. Monometallic catalysts with the same weight loadings as the 1%Ru−2.5%Mo/CNT, similar bimetallic catalysts supported on alumina and silica, and a higher loading bimetallic (5%Ru−20%Mo/CNT) catalyst were also prepared for characterization comparisons.

Catalytic reaction of furfural to cyclopentanol was carried out in a 50 ml autoclave (Anhui Kemi Machinery Technology Co., Ltd.) with magnetic stirring. In a typical experiment, 0.05 g catalyst, 0.25 g furfural, and 5 ml H_2_O were added into the autoclave first. The autoclave was then sealed, flushed with low pressure hydrogen six times, and pressurized with hydrogen to 4.0 MPa. Finally, the autoclave was heated to the desired temperature and maintained there for a set reaction time (0.5–8 h). The reactor was then quickly cooled, the reaction mixture was poured to a vial (20 ml), and the catalyst was separated from the aqueous phase by centrifugation.

The liquid products were analyzed by gas chromatography (Varian DB-WAX capillary column, 30 m × 0.320 mm × 0.50 μm) equipped with a flame ionization detector (FID) via internal standard method by adding a certain amount of N, N-dimethylformamide (DMF) to the vial as the internal standard. Moreover, gas chromatography-mass spectroscopy (GC-MS, Thermo Trace GC Ultra with a Polaris-Q ion trap mass spectrometer) equipped with a VF-17MS capillary column was applied to analyze the species in liquid samples. The conversion of furfural was calculated on the ratio of converted furfural to fed furfural, while the selectivity to a given product was evaluated as the ratio of the yield of that product to converted furfural.

N_2_ adsorption–desorption isotherms of the catalysts and the support were measured using a QUADRASORB SI analyzer equipped with a QuadraWin software system at liquid nitrogen temperature. Prior to measurements, the samples were degassed at 150°C for 12 h under vacuum. The apparent surface area (A_BET_) of carbon materials were calculated using the Brunauer–Emmett–Teller equation. The crystalline structure of the catalysts was characterized by X-ray diffraction (XRD) (Rigaku Co., Japan, Cu Kα radiation (λ = 0.154 nm)) operated at 40 kV and 200 mA. XPS analyses of the samples were obtained using a Leybold LH 10 spectrometer equipped with a single-channel detector, employing Al Ka radiation (1253.6 eV, anode operated at 10 KV X 23 mA). The C1s peak position was set at 284.4 eV and used as reference to locate the other peaks. The microstructure and metal distribution were characterized by transmission electron microcopy (TEM) with a JEOL JEM2010F transmission electron microscope equipped with a field emission gun emitter. Hydrogen temperature programmed reduction (H_2_-TPR) was conducted on a Micromeritics ChemiSorb 2720 with a thermal conductivity detector (TCD). In a typical experiment, a certain amount of catalyst was placed in a U-tube quartz reactor followed by treatment at 150°C with flowing He (50 cm^3^/min) for 2 h. The sample was then cooled to room temperature and maintained for 1 h, followed by switching the feed from He to 10% H_2_/Ar (30 cm^3^/min), and increasing the temperature to 900°C at a heating rate of 10°C/min. Finally, NH_3_ temperature-programmed desorption (NH_3_-TPD) with a Micromeritics ChemiSorb 2750 pulse chemisorption system was used to estimate the acidity. First, the sample was pre-treated under pure hydrogen at different temperatures to reduce the Ru-Mo/CNT precursors *in-situ*. Second, the feed was switched from hydrogen to helium flow (30 cm^3^/min) while the sample temperature was increased to 400°C for 1 h to eliminate impurities on the catalyst surface. Then, the sample was cooled to 50°C and exposed to ammonia in a stream of 10% NH_3_/He flow (50 ml/min) for 1 h. Finally, desorption of NH_3_ was carried out in a flow of helium (30 cm^3^/min) as the temperature was increased from room temperature to 800°C at a heating rate of 10°C/min.

## Results and Discussion

### Catalytic Activity Tests

Screening experiments over the monometallic Ru/CNT and Mo/CNT catalyst, as well as similar bimetallic catalysts on different supports (Ru-Mo/CNT, Ru-Mo/SiO2, Ru-Mo/Al2O3, Ru-Mo/AC) were carried out. As shown in Table 1, for most catalysts, the feedstock furfural was completely converted and the main products were furfural alcohol (FAL), tetrahydrofurfuryl alcohol (THFA), cyclopentanone (CPO) and cyclopentanol (CPL). (The GC trace was supported in the Supplementary Figure 1) Besides the products named above, small amounts of 2-cyclopetenone, 2-methylfuran, 5-hydroxy-2pentanone, 1, 2-pentanediol, 1, 5-pentanediol, and 1-pentanol were detected in the products. When comparing all catalysts, most catalysts displayed inferior performance to that of Ru-Mo/CNT catalyst, which demonstrated completed furfural conversion and 81.0% yield of cyclopentanol product at an optimized reaction condition. Moreover, the investigation of the proportion of bimetallic components suggested that an increase in the ratio of Ru and Mo content in the catalyst was conducive to the production of tetrahydrofurfuryl alcohol (THFA), but it is difficult to continue the rearrangement reaction according to the relevant literatures (Zhang et al., 2016, 2019; Mironenko et al., 2018). In addition, the increase in the proportion of Mo content was beneficial to the rearrangement of the products, whereby 97.2% total yield of CPO and CPL were obtained over 1%-Ru−2.5%Mo/CNT more than that of 1%Ru−1%Mo/CNT. As explained in a previous work, the catalytic performance could be attributed to the synergistic effect on bimetallic Ru-Mo catalysts and the special electronic properties of carbon nanotubes (Wang et al., 2019).

**Table 1:** Influence of reaction conditions to the conversion of FFA.

**Entry**	**Catalysts**	**Conv. (%)**	**Yield (C**_****mol****_ **%)**			
			**FAL^**a**^**	**THFA^**a**^**	**CPO^**a**^**	**CPL^**a**^**
1	1%Ru/CNT	100	–	35.4	–	10.9
2	2.5%Mo/CNT	42.0	8.1	–	–	–
3	1%Ru-2.5%Mo/CNT	100	–	–	–	81.0
4	1%Ru-1%Mo/CNT	100	–	47.3	–	20.7
5	1%Ru-5%Mo/CNT	100	–	50.6	–	38.9
6	5%Ru-20%Mo/CNT	100	–	6.8	25.3	64.3
7	1%Ru-2.5%Mo/SiO_2_	100	–	–	3.7	44.0
8	1%Ru-2.5%Mo/Al_2_O_3_	61.0	17.5	7.9	21.7	10.6
9	1%Ru-2.5%Mo/AC	100	–	7.4	–	44.0

a FAL, furfural alcohol; THFA, tetrahydrofurfuryl alcohol; CPO, cyclopentanone; CPL, cyclopentanol.

*Reaction condition, 0.05 g catalyst (all catalysts reduced at 600°C), 0.25 g furfural, 160°C, 4 MPa H_2_ pressure, 1,000 rpm stirring speed, 5 ml water, for 4 h. “–” indicates a trace amount*.

To optimize the result, reaction conditions were also studied in detail (Table 2). According to the literature, the hydrogenation rearrangement of furfural into cyclopentanol involves multistep reactions with different energy barriers (Banerjee and Mushrif, 2017). Thus, the product distribution is significantly affected by reaction temperatures. At a low reaction temperature (120°C), FFA (65.4%) was the main product because the active aldehyde group can be easily hydrogenated (Deng et al., 2018). As the reaction temperature increased, the amount of rearrangement products (CPO and CPL) increased, with 81.0 and 89.1% yields of CPL obtained at temperatures of 160 and 180°C, respectively. However, the CPL yield decreased to 72.9% when the reaction temperature was further increased to 200°C. The effect of H_2_ pressure was also investigated, with high amounts of FAL and CPO products (inadequately hydrogenated products with unsaturated C=C and C=O bonds) detected for the reaction at low H_2_ pressure (2 MPa) and low temperature (140°C, entry 2 in Table 2). Conversely, high amounts of THFA (an over-hydrogenated products with saturated bonds) were detected for the reaction at high H_2_ pressure (6 MPa) and high temperature (180°C, entry 7 in Table 2). Moreover, an experiment with high concentration substrate was also carried out, but showed poor performance with a 54.4% yield of CPL, indicating that high concentration was not conducive to the production of CPL. These indicated that the hydrogenation performance is significantly influenced by the reaction conditions.

**Table 2:** Influence of reaction conditions to the conversion of FFA.

**Entry^**a**^**	**Temp.** **(**^**°**^**C)**	**Pressure** **(MPa)**	**Conv.** **(%)**	**Yield (C**_****mol****_ **%)**			
				**FAL^**b**^**	**THFA^**b**^**	**CPO^**b**^**	**CPL^**b**^**
1	120	4	100	65.4	-	4.0	2.6
2	140	2	100	35.8	8.9	17.5	31.2
3	160	4	100	–	–	–	81.0
4	180	4	100	–	–	–	89.1
5	200	4	100	–	–	–	72.9
6	180	1	100	–	–	47.6	33.5
7	180	6	100	–	23.0	–	49.2
8^c^	160	4	100	–	27.1	–	54.4

a 1% Ru-2.5%Mo/CNT,

b FAL: furfural alcohol, THFA, tetrahydrofurfuryl alcohol; CPO, cyclopentanone; CPL, cyclopentanol.

c *All other conditions are same to 3#, but with 0.5 g furfural in 5 mL water. Reaction condition: 0.05 g catalyst (all catalysts reduced at 600°C), 0.25 g furfural, 1,000 rpm stirring speed, 5 ml water, for 4 h. “–” indicates a trace amount*.

**Figure 1:**
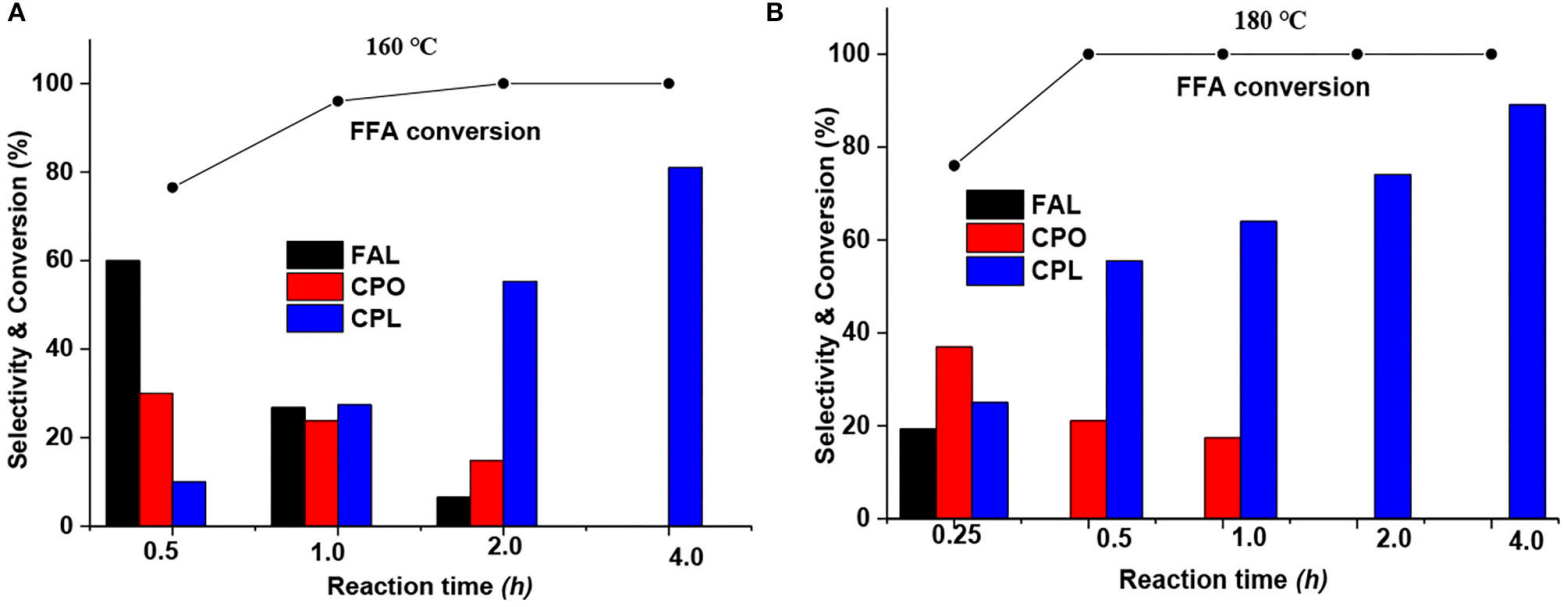
Influence of reaction time at reaction temperatures of **(A)** 160°C and **(B)** 180°C. Reaction condition: 1% Ru−2.5%Mo/CNT, 0.05 g catalyst (reduced at 600°C), 0.25 g furfural, 1,000 rpm stirring speed, 5 ml water, 4 MPa (H_2_). Solid curves indicated the FFA conversion trends.

Moreover, the influence of the reaction time at optimized reaction temperatures of 160 and 180°C was also investigated. As shown in Figure 1A, furfural hydrogenation at 160°C displayed a gradual change in product distribution at different reaction times. For instance, the selectivity to FAL was reduced from 60 to 27 to 7% as the reaction time was increased from 0.5 to 1.0 to 2.0 h. The selectivity to CPO decreased from 35 to 24 to 14% at the reaction time of 0.5, 1.0 to 2.0 h. The selectivity of CPL gradually increased from an initial 5 to 81% as the reaction time increased from 0.5 to 4.0 h. In contrast, the product distribution at 180°C showed a relatively rapid change with reaction time. As shown in Figure 1B, no FAL product was detected at a reaction time of 0.5 h. Thus, a test at a shorter reaction time (0.25 h) was carried out, which gave a 19% FAL product selectivity, reflecting that the reaction rate was greatly promoted at high reaction temperatures. Meanwhile, the selectivity to CPO dropped from 37% at 0.25 h to 0% at 2 h, while the amount of CPL increased to 89% as the reaction time was increased to 4 h. In general, CPL is the series product of CPO hydrogenation. But it is still hard to get high yields of CPL product. Hronec et al. studied the influence of furanic polymers (formed via the condensation reaction on acid sites) on the selectivity of furfural rearrangement and found that the deposition of polymers on the catalyst surface can negatively influence its activity, in particular by inhibiting the consecutive hydrogenation of CPO to CPL (Hronec et al., 2013).

**Figure 2:**
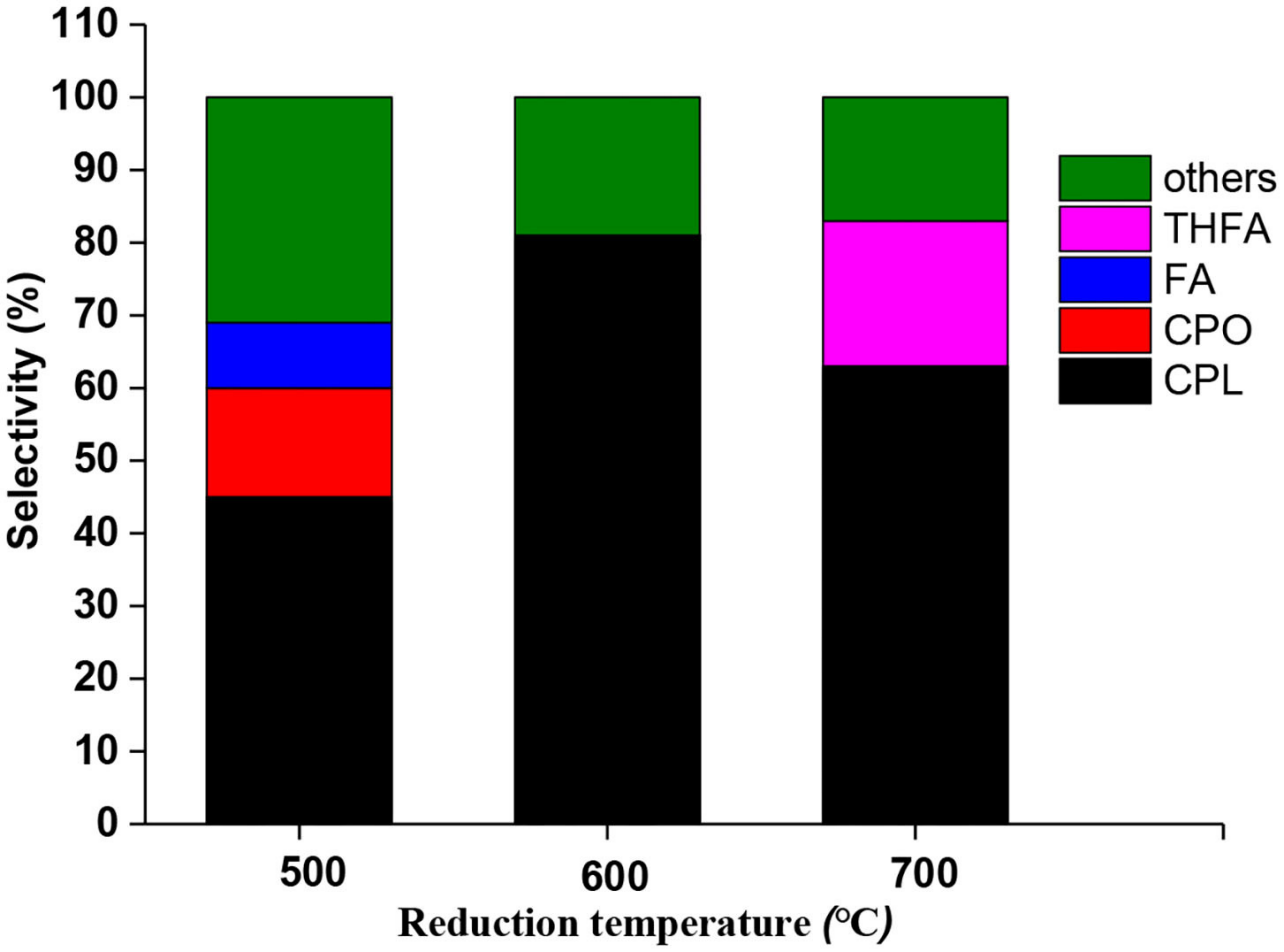
Product distribution of Ru-Mo catalyst after treatment at different reduction temperatures. Reaction condition: 0.05 g catalyst (1% Ru-2.5%Mo/CNT), 0.25 g furfural, 1,000 rpm stirring speed, 5 ml water, 160°C, 4 MPa (H_2_).

Since reduction temperature played a very critical role in catalyst activation, a comparison test was also conducted among catalysts prepared at different reduction temperatures. As shown in Figure 2, these catalysts displayed different product distributions. Catalyst reduced at 500°C showed a low CPL selectivity (46%) and partial intermediate selectivity, but catalyst reduced at 700°C showed a selectivity to over-hydrogenated product of THFA. The catalyst 600°C showed maximum CPL selectivity (81%), indicating that it is the best one for hydrogenation-rearrangement reaction. Moreover, Xu et al. pointed out that the weak acid or neutral sites would be more favorable to the rearrangement of furfural than the strong acidic sites (Yang et al., 2013; Zhang et al., 2019). In fact, unsaturated furfural compounds were easily polymerized on acidic sites (Wang et al., 2019). Therefore, it is necessary to regulate the acid sites or protons to avoid undesirable polymerization by-products.

Catalyst stability would be challenging in a hydrothermal environment. Here, a recycling experiment was also conducted to evaluate the extent of deactivation during the reaction. Prior to each reuse of Ru-Mo catalyst, the reaction crude was allowed to settle down, and the supernatant was removed from the reactor. Then, a fresh charge of reactant was added to the reactor and the subsequent run was continued. As shown in Supplementary Figure 2, no deactivation of catalyst was observed in the first two recycle runs with CPL selectivity maintained above 80%, while the recycling experiment of run three had shown a tendency of deactivation with a CPL selectivity lower than 80%. Moreover, characterization on the recycled catalyst was also carried out. The XRD pattern showed a peak broadening of the spent catalyst (Supplementary Figure S3A) and TEM presented that nanoparticles were relatively uniformly distributed on spent catalyst (average size: 1.8 nm, Supplementary Figure S3B inset). It is noted that the catalyst was repeatedly exposed to the hydrothermal environment without a regenerated process. Thus, the catalyst would be more effective with a properly regenerated process by hydrogen treatment after reaction (Deng et al., 2018).

### Characterization Analysis and Mechanism Study

**Figure 3:**
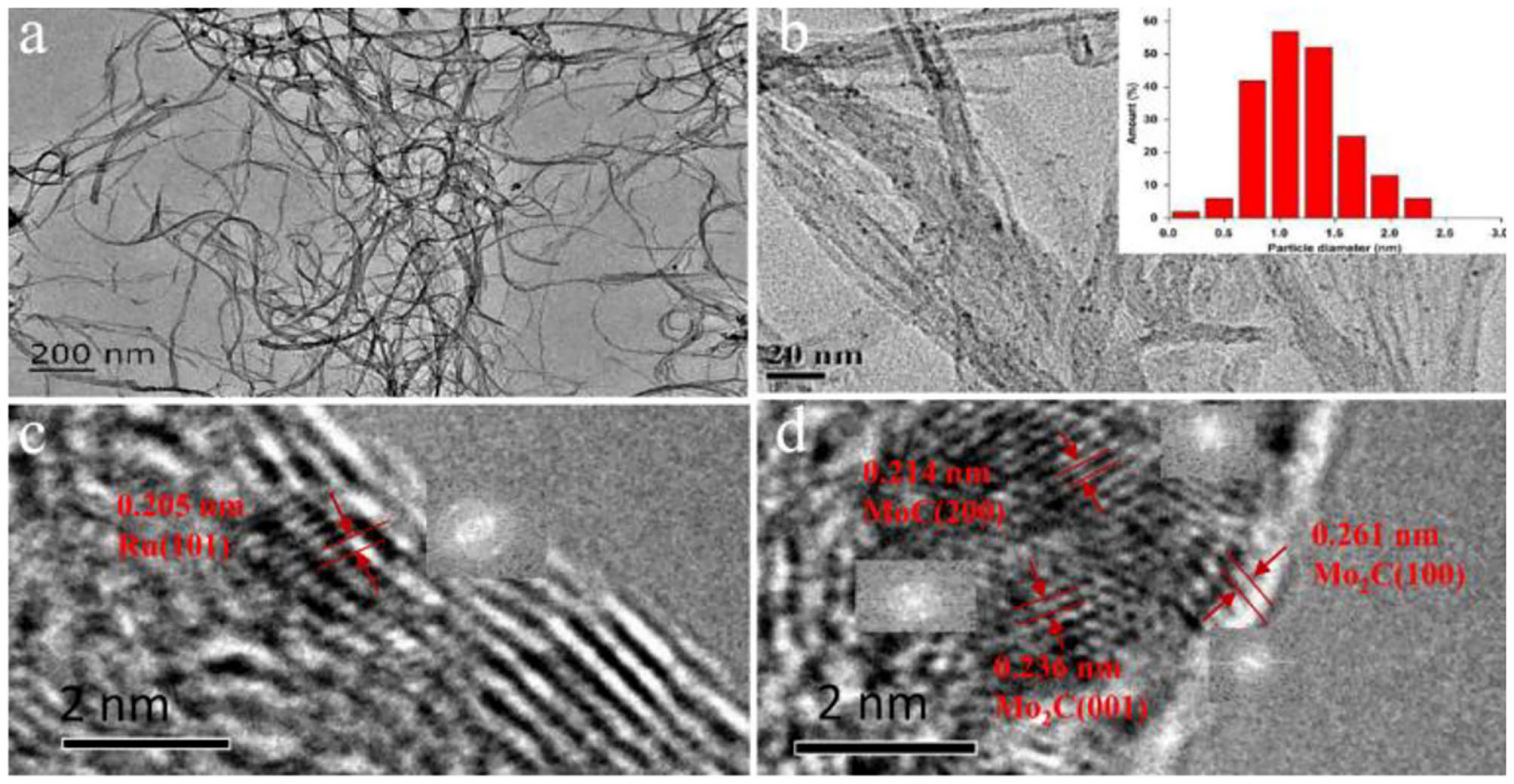
**(a,b)** TEM and **(c,d)** HRTEM images of 1%Ru-2.5%Mo/CNT catalyst.

Supplementary Figure 4 showed the N_2_ adsorption-desorption results of the CNT and 1%Ru−2.5%Mo/CNT samples. Both possessed hysteresis loops due to the mesoscale porosity, but have no knees at low relative pressure, suggesting little microporous structure. The surface area and pore volume were respectively 231 m^2^/g and 1.78 cm^3^/g for the unimpregnated CNT, which were slightly reduced to 228.3 m^2^/g or unchanged at 1.78 cm^3^/g for the impregnated Ru-Mo/CNT catalyst. The pore width distribution of the samples showed mesopores in a wide range of 2–30 nm and minimal micropore distribution, which indicates that the nanotubes remained intact after impregnation and reaction processes. TEM and corresponding HRTEM images were used to study the morphology and microstructure of the 1%Ru−2.5%Mo/CNT catalyst (Figure 3). Most of the carbon nanotubes retained intact structures and uniform sizes, with outer diameters in the range of 5–8 nm and inner diameters 2–5 nm. Moreover, the metal nanoparticles were well-dispersed on the outer surface of the carbon nanotubes, with an average particle size of 1.4 nm (Figure 3b inset) (Sun et al., 2017). No significant formation of aggregates was detected (Hanelt et al., 2011; Aftab et al., 2013). In addition, HRTEM images with insets of fast Fourier transform (FFT) patterns of typical nanoparticles were also employed to identify the nanostructures. As shown in Figure 3c, lattice fringes with interplanar spacing of 0.205 nm corresponded to (101) facet of metal Ru^0^ particles (Wang et al., 2014; Zhu et al., 2017) were observed. Another nearby nanoparticle (Figure 3d) was displayed lattice fringes of 0.214 nm, corresponding to the (200) facet of MoC, and 0.236 and 0.261 nm, corresponding to the (001) and (100) facets of Mo_2_C, respectively (Li J. et al., 2014; Grilc et al., 2015). In addition, some amorphous or poorly crystallized nanoparticles were also detected on the surface, which may be ascribed to the Mo-based amorphous species (MoO_x_, MoC_x_, MoC_x_O_y_, etc.) (Pascale Delporte et al., 1995; Wang et al., 2015; Zhang et al., 2017).

**Figure 4:**
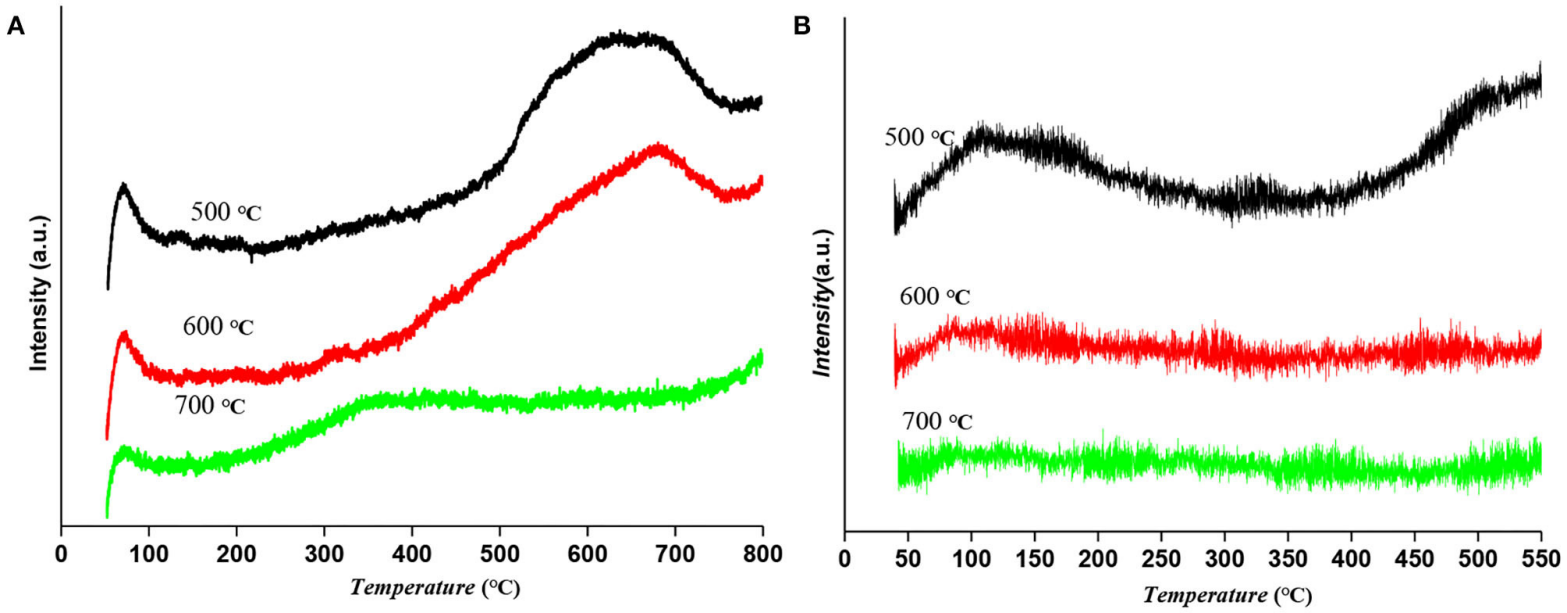
**(A)** H_2_-TPD and **(B)** NH_3_-TPD result of Ru-Mo/CNT catalyst reduced at different temperatures.

H_2_-TPD of Ru-Mo/CNT catalysts reduced at different temperatures were also investigated to study the change of metal sites. As shown in Figure 4A, the catalyst reduced at low temperature had a low temperature hydrogen desorption peak (50–120°C) and a high temperature hydrogen desorption peak (500–700°C), while the high temperature hydrogen desorption peak was gradually weakened when the reduction temperature increased. Interestingly, the high temperature peak was disappeared and a broad moderate desorption peak at around 300°C was appeared on the catalyst reduced at 700°C. These results suggested that the catalyst reduction temperature has a great influence on the hydrogen adsorption sites on the catalyst, especially the strong hydrogen adsorption sites. According to the relevant literature, one of the characteristics of Ru catalyst is the strong hydrogen adsorption phenomenon, which is conducive to hydrogenation in gas-liquid-solid polyphase reactions (Li et al., 2013; Chen et al., 2014). Moreover, NH_3_-TPD was carried out to determine the surface acidity. As shown in Figure 4B, the low temperature peaks (100–250°C) are attributed to the NH_3_ desorption from weak acidic sites, whereas the high temperature peaks (>250°C) are ascribed to NH_3_ desorption from medium-strength acidic sites. The catalyst reduced at 500°C gave a broad NH_3_ desorption peak at a low temperature, but for catalysts at 700°C reduction temperatures, the intensity NH_3_ desorption peaks had almost no acidity due to the presence of molybdenum carbide. Interestingly, the result of the catalyst reduced at 600°C showed weakly acidic compared to that of the catalyst reduced at 500°C, which could be attributed to the Lewis acidity of molybdenum oxycarbide (MoO_x_C_y_) species, an isomerization catalytic site with relatively low oxophilicity and Lewis acidity according to the previous reports.

**Figure 5:**
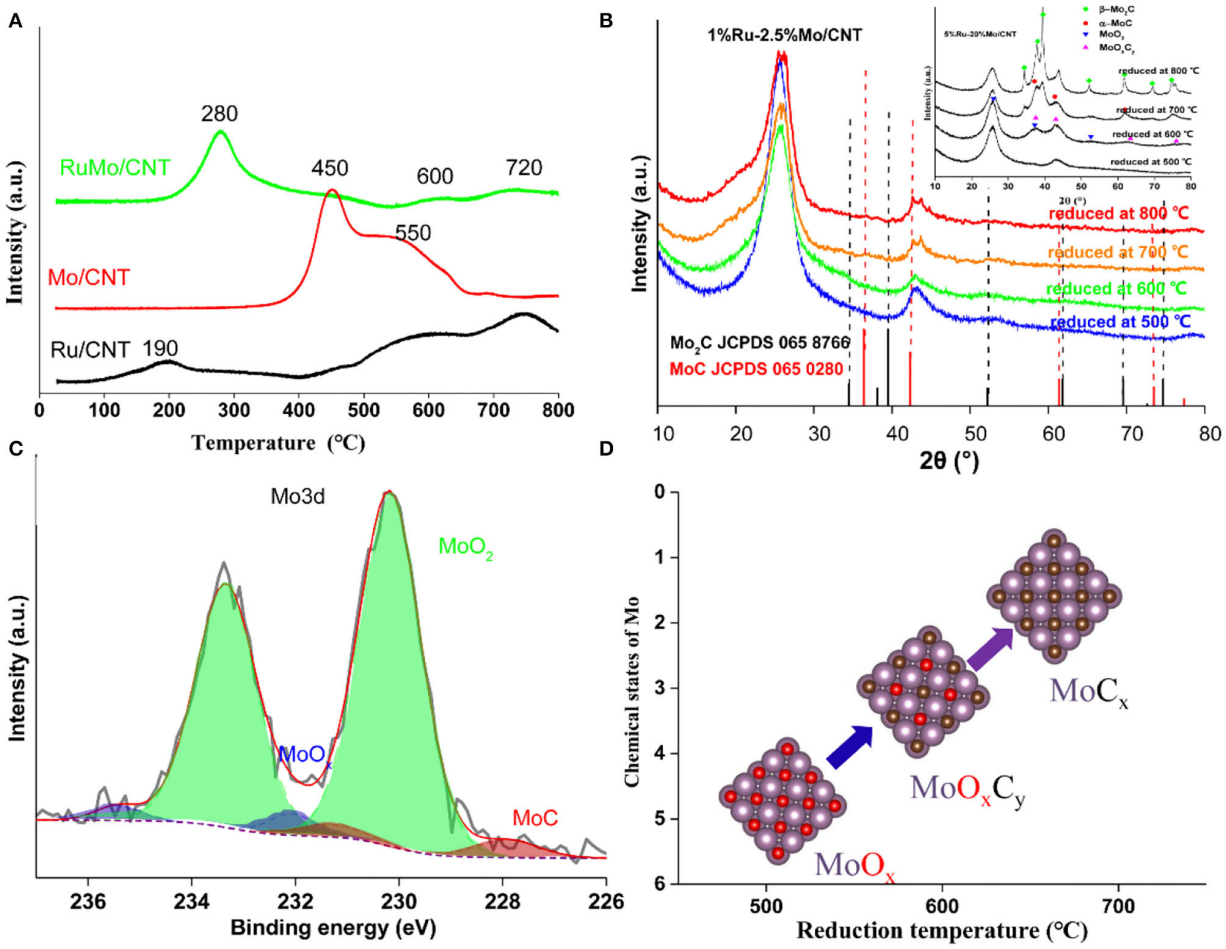
**(A)** H_2_-TPR of typical catalysts (1%Ru/CNT, 2.5%Mo/CNT, 1%Ru-2.5Mo%/CNT), **(B)** XRD patterns of Ru-Mo/CNT catalysts reduced at different temperatures, **(C)** XPS spectra of the Mo3d region of the 1%Ru-2.5%Mo/CNT catalyst, and **(D)** Schematic illustration of Mo species transformation.

The H_2_-TPR experiment displayed the reduction performance of catalysts. As shown in Figure 5A, the reduction peak of monometallic Ru catalyst occurred at around 200°C, while the reduction of monometallic Mo catalyst occurred above 400°C. However, the main reduction peak present on 1%Ru-2.5%Mo/CNT catalyst occurred at about 280°C. The different reduction behavior is attributed to the bimetallic Ru-MoO_x_ structures on the catalyst, which is interpreted as Ru facilitating the reduction of nearby MoO_x_ species due to the hydrogen spillover from the Ru sites. Moreover, several small reduction peaks at around 460, 600, and 720°C were also observed on Ru-Mo/CNT catalyst, which are likely due to the different reduction stages of the various Mo species. Figure 5B showed the XRD patterns of 1%Ru−2.5%Mo/CNT catalyst at different reduction temperatures. As expected, two diffraction peaks due to carbon at around 26 and 44° (graphite C JCPDS 01-075-162) are apparent; however, no obvious peaks associated with metallic Ru or Mo oxide species were detected due to the low loading amount and/or the good dispersion of the small nanoparticles. For comparison, an XRD pattern of a high metal loading catalyst (5%Ru-20%Mo/CNT) is shown in the inset of Figure 5B, which suggested that the catalyst reduced at 500°C displayed an amorphous character, diffraction peaks at 26.0, 36.9 and 53.4° appeared at a reduction temperature of 600°C, which are attributed to MoO_2_ (JCPDS 78-1070), and molybdenum carbide crystal phases (α-MoC (JCPDS 65-0280, 2θ=36.4, 42.4, and 61.6°) and β-Mo_2_C (JCPDS 35-0787, 2θ=34.4, 38.0, 39.4, 52.1, 61.5, and 69.6°) were detected on the support at the temperature above 700°C (Liang et al., 2017; Posada-Pérez et al., 2017; Wang et al., 2017). These results indicated the transformation of Mo species from molybdenum oxide to molybdenum carbide during the reduction and carburization process (Iida et al., 2017; Liang et al., 2017; Posada-Pérez et al., 2017; Gao et al., 2018; Kou et al., 2018). XPS was carried out to identify the surface chemical state of the 1%Ru−2.5%Mo/CNT catalyst reduced at 600°C. As shown in Figure 5C, it was deconvoluted into three doublets: at 228, 230, and 232 eV, which can be attributed to different Mo species of MoC, MoO_2_, and MoO_x_, respectively (Chen et al., 2015; Zhang et al., 2017). These surface species of coordinatively unsaturated metal centers (Mo^2+^, Mo^4+^, and Mo^5+^) were reported to account for the catalytic performance (Li Z. et al., 2014). Moreover, XPS profiles of Mo3d of different temperature reduced catalysts were also supported for a comparison study, which showed that a slight shift to low binding energy for catalysts reduced at high temperatures (Supplementary Figure 5).

Taken all together, the above results indicated that the RuMo bimetallic catalyst reduced at 600°C presented a synergistic effect between the weak acidity and strong hydrogenation sites for the hydrogenation-rearrangement of biomass furans compounds. Different to the monometallic catalysts, the bimetallic catalysts ensured the higher efficiency of H_2_-sippover and interaction between Ru and MoO_x_ species in vicinity according to literatures (Weng et al., 2018a,b), thus the bimetallic catalyst showed closer correlation during catalyst reduction and carburization. According to the characterization results, the reduction of molybdenum species in the catalyst was promoted due to H_2_-sippover, and the active phase of molybdenum carbide as well as other Mo-based amorphous species (MoO_x_, MoC_x_, MoC_x_O_y_, etc.) appeared in the catalyst at 600°C, which also changed the strength and content of the metal and acid sites on the catalyst surface. Especially, molybdenum oxycarbide (MoO_x_C_y_) species, a transitional form during the dynamic transformation, may occur, which was a very good isomerization catalyst for alkanes. As for the *in-situ* analysis for the aqueous reaction, which is challenging at present, the detailed catalytic mechanism of ruthenium-molybdenum (oxy) carbides catalysts will be investigated in our future work.

## Conclusions

In this work, we reported the design of a low loading bimetallic Ru-Mo/CNT catalyst for the hydrogenation-rearrangement reaction of furfural into cyclopentanol with high selectivity in the aqueous phase. The results indicated that the selectivity to rearrangement products is highly sensitive to both the reaction conditions (reaction temperature, H_2_ pressure, contact time, etc.) and to the formulation of the catalysts. Moreover, it also suggested that the catalytic performance of Ru-Mo/CNT catalyst could be tuned by controlling the reduction and carburization degree to retain the weak Lewis acidity and strong hydrogenation activity. These findings provide a convenient strategy to tune the catalytic performance of Mo-based catalysts by controlling the reduction and carburization conditions, which appear to be versatile for the rearrangement of furans and similar compounds.

## Data Availability Statement

The original contributions generated for the study are included in the article/Supplementary Materials, further inquiries can be directed to the corresponding author/s.

## Author Contributions

YW, QS, and YZ designed the experiments. SM, XW, HY, and ZW conducted the experiments. YW and MF analyzed the data. SM and YW wrote the first draft of the manuscript which was then revised by all other authors.

## Conflict of Interest

The authors declare that the research was conducted in the absence of any commercial or financial relationships that could be construed as a potential conflict of interest.
