# Supplementary material for: Renewable Cyclopentanol From Catalytic Hydrogenation-Rearrangement of Biomass Furfural Over Ruthenium-Molybdenum Bimetallic Catalysts
Source: Front Bioeng Biotechnol. 2020 Dec 18;8:615235. doi: 10.3389/fbioe.2020.615235 (PMC7775491; doi:10.3389/fbioe.2020.615235)
Supplement: Supplementary file 1 [file Table_1.DOCX]

**Supplemental material.**

Fig. S1 GC trace of RuMo/CNT catalyst.

Fig. S2 Recycling test of RuMo catalyst in the conversion of FFA, Reaction condition: 1% Ru-2.5%Mo/CNT, 0.05g catalyst (reduced at 600 ℃), 0.25g furfural, 1000 rpm, 5 ml water, 4 MPa (H_2_).


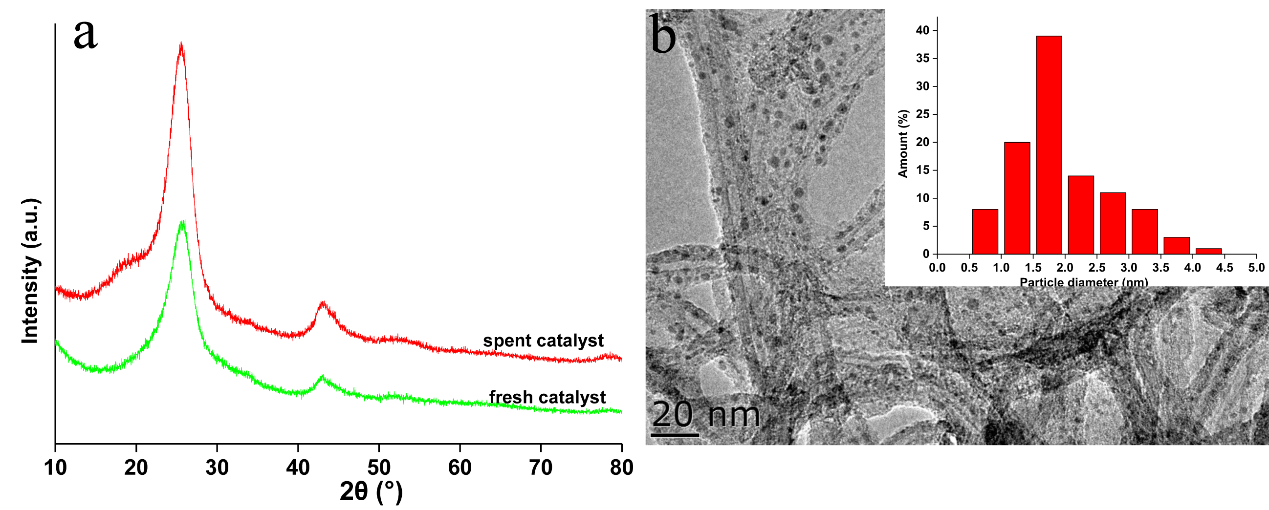


Fig.S3 (a) XRD pattern and (b) TEM images of spent 1% Ru-2.5%Mo/CNT after the recycling experiment.

Fig. S4 (a) N_2_ adsorption-desorption isotherms at 77K, (b) Pore size distribution curves.

Fig. S5 XPS result of Ru-Mo/CNT catalysts reduced at different temperatures.
